# Why Govern Broken Tools?

**DOI:** 10.1017/jme.2023.21

**Published:** 2022

**Authors:** Ryan Calo

**Affiliations:** 1. UNIVERSITY OF WASHINGTON SCHOOL OF LAW, SEATTLE, WA, USA

**Keywords:** Digital Contact Tracing, COVID, Governance Critique

## Abstract

In *Assessing the Governance of Digital Contact Tracing in Response to COVID-19: Results of a Multi-National Study*, Brian Hutler et al. ably compare two approaches to the governance of digital contract tracing (DCT).^1^ In this brief essay, I want to examine to what extent governance actually played a meaningful role in the failure of DCT. If DCT failed primarily for other reasons, then the authors’ normative suggestion to pursue “a new governance approach … for designing and implementing DCT technology going forward” may be misplaced.

In *Assessing the Governance of Digital Contact Tracing in Response to COVID-19: Results of a Multi-National Study*, Brian Hutler et al. ably compare two approaches to the governance of digital contract tracing (DCT).^1^ In this brief essay, I want to examine to what extent governance actually played a meaningful role in the failure of DCT. If DCT failed primarily for other reasons, then the authors’ normative suggestion to pursue “a new governance approach … for designing and implementing DCT technology going forward” may be misplaced.

The study’s authors define DCT broadly as a “category of technologies used to facilitate the identification of individuals potentially exposed to disease agents,” but quickly make it clear that the study specifically focuses on the use of mobile phones to alert participants to potential COVID exposure. The first approach to governance, deployed by certain European and American regulators, centers privacy and data protection. The second, deployed by Israel, South Korea, Ghana, and South Africa, prioritizes emergency response. Neither approach best services the goals of DCT, the study’s authors argue. The data protection model threatens the efficacy of DCT by limiting its functionality and, ironically, scaring away participants. The emergency response model sacrifices civil liberties at the altar of public health.

DCT as deployed today, using Bluetooth technology for a novel purpose, faces many technical obstacles. The system works by detecting sufficient proximity — a short enough distance for a long enough time — to the phone of a person who later reports having COVID. Opportunities for false positives and negatives abound. A participant could be notified of exposure to a friend they met outside while wearing masks, or even to their neighbor in the next apartment (since Bluetooth travels through walls). Alternatively, a participant could sit next to an infected individual on a plane whose phone happens to be in airplane mode or speak face to face with a delivery person who left their phone in the car.

Setting the proper threshold for contact, meanwhile, is daunting. Researchers do not know exactly how long it takes to catch COVID and, unlike human contact tracers, the DCT system does not use follow up questions to gain additional context. Meanwhile, the virus is always changing: reasonable parameters (six feet for fifteen minutes, for example) set for the Alpha or Delta strain are likely to prove woefully inadequate for Omicron.

We should not be surprised, therefore, that the evidence that DCT actually arrests the spread of COVID is limited. As the authors readily acknowledge, only a small handful of studies, some of which were undertaken by the same people who built the applications under examination, exist to demonstrate efficacy. They do so largely by deploying statistical models to estimate the number of participants who tested positive and quarantined as a consequence of receiving an automated exposure alert, sometimes in comparison to human or no contact tracing. The assumption is that this phenomenon of true positives slowed the spread of COVID in participating jurisdictions.

My review of these studies left me with questions. Where community transmission is high, would not *randomly* notifying people of exposure save lives too by prompting some coincidentally asymptomatic carriers to get tested? And in a world of finite resources, should we not place false positives leading to unnecessary testing, let alone the significant costs of DCT systems themselves, on the other side of the public health ledger?

In other words, Hutler et al.’s conclusion only follows if one concedes an important and contested assumption. The proper response to the failures of DCT may not be a better balance of privacy, civic participation, and emergency response. It may be to invest scarce public dollars elsewhere.

I was part of a team at the University of Washington studying public privacy attitudes about contract tracing. We found privacy and security concerns in our surveys that may have, in theory, dampened participation in DCT and compromised their performance.^2^ And yet, according to the Hutler et al., most empirical studies show that “members of the public cite lack of perceived benefit as the main reason for not using DCT apps.” These members of the public may be on to something.

I agree with Hutler et al. regarding the imperfections of data protection and emergency response. Both models go too far in their way. Part of the reason that we do not have good data on the efficacy of DCT is that many apps were designed to resist centralized analysis. But if it turns out that Bluetooth-enabled contact tracing amounts to a lot of noise, as you might expect when a technology is being used in a way it was not designed for; if it turns out that that randomized testing, vaccine awareness campaigns, or other investments would have better served public health; then it is not clear why societies should attempt to find a Goldilocks form of governance for DCT at all.

In other words, Hutler et al.’s conclusion only follows if one concedes an important and contested assumption. The proper response to the failures of DCT may not be a better balance of privacy, civic participation, and emergency response. It may be to invest scarce public dollars elsewhere.
